# The Assessment of Dyspnea during the Vigorous Intensity Exercise by Three Dyspnea Rating Scales in Inactive Medical Personnel

**DOI:** 10.5539/gjhs.v5n6p19

**Published:** 2013-07-24

**Authors:** Patrawut Intarakamhang, Piyathida Wangjongmeechaikul

**Affiliations:** 1 Department of Physical Medicine and Rehabilitation, Phramongkutklao College of medicine and Hospital, Bangkok, Thailand

**Keywords:** dyspnea rating scale, exercise, vigorous intensity, Borg scale, Numerical Rating Scale, FACES, Likert scale

## Abstract

It is well recognized that exercise is good for health especially as it’s known to prevent metabolic syndromes such as diabetes, hypertension and heart disease. To reap the benefits from exercise the most appropriate level of intensity must be determined, the level of intensity ranging from low, low to moderate to hard (vigorous).

This study is aimed to 1. To investigate and evaluate 3 subjective rating scales. The Borg scale, the Combined Numerical Rating Scale (NRS) + FACES Dyspnea Rating Scale (FACES) and the Likert scale, during hard (vigorous) exercise. 2. To compare the effectiveness of the Borg scale and Combined Numerical Rating Scale (NRS) + FACES Dyspnea Rating Scale during the hard (vigorous) intensity exercise. This study uses a descriptive methodology.

The sample group was 73 medical personnel that were leading an inactive life style, volunteers from Phramongkutklao Hospital. Participants were randomly divided into 3 groups. Group 1, those to report using the Borg Scale, group 2 using NRS + FACES, and group 3 to subjectively assess the intensity of the exercise using the Likert scale during a treadmill Exercise Stress Test (EST) using the Bruce protocol. The upper limit of the intensity in the study was equal to 85% of the maximal heart rate of all participants. The subjective reporting of the experienced level of dyspnea was undertaken immediately after the completion of exercise.

The average age of participants was 23.37 years old. The 26 participants reporting using the Borg scale had mean Borg scale score of 13.46+1.77, a mode score of 15. The 24 participants reporting intensity levels through NRS +FACES had a mean NRS + FACES score of 6.83+1.09 and mode on the NRS + FACES scale equal to 7. The Likert scale group evaluated 23 participants with a mean Likert scale score of 2.74. That is those choosing Levels 2 and 3 were 6 (26.9%) and 17 participants (73.95%), respectively. Comparing the two groups with the Borg scale at equal to or greater than 15 and NRS + FACES greater than or equal to 7 using a Chi-square test showed that there were no statistical significant differences at *p* = 0.084. Using Spearman’s rank correlation coefficient it was found that the subjective rating of intensity in the 3 different groups was not statistically significantly related to heart rate at 85% of maximal heart rate (*P*> 0.05). NRS + FACES, the evaluation of the intensity of exercise at the hard (vigorous) level, was not statistically significantly different from the Borg scale.

## 1. Introduction

Exercise contributes to health benefits by controlling diseases in the metabolic syndrome group, including diabetes, hypertension, lipid disorders and obesity. It is helps to prevent and reduce the mortality rate from coronary heart disease and decreases the risk of falling in the elderly (Pollock et al., 1998; Mazzeo, 1998; Laskowski et al., 2012). There are 4 important components of prescription for exercise to be effective (Pollock et al., 1998; Phillips et al., 2012). These are known by the acronym “FITT”, F = frequency, I = intensity, T =Type and another T = time or duration. In the literature many recommendations have been made and accepted. The Review of Physical Activity Recommendation in 1995, requires that it is necessary to “accumulate 30 minutes of moderate physical activity on most days.” which has later been adjusted to include vigorous (hard) intensity aerobic, physical activity. It was recommended that to reach the moderate/vigorous level people should accumulated 150/75 minutes per week, and increase to 300/150 minutes, respectively (Oja et al., 2010) to obtain the additional benefits.

The Overload principle says to reap the health benefits from exercise it is important to have an optimum level of intensity that is suitable for minimum duration of exercise each other (Laskowski et al., 2012). Thus these 2 components, intensity and duration are inversely proportional (Pollock et al., 1998). The Physical Activity Guideline for Americans suggests that to achieve the minimum goal of exercise for health, that should be accomplished by moderate intensity, aerobic exercise while the Center of Disease and Control (CDC) recommends that “Low to moderate intensity aerobic exercise can and should be given to most people”. In case of the heavy intensity exercise, the exerciser should be carefully considered and evaluated himself before starting the activity (Phillips et al., 2012).

Among 4 components of exercise prescription, the intensity seems to be the most important leading factor because the risks of complications (adverse events) may occur during exercise and they are exactly associated with the level of intensity. For healthy people those complications such as sudden cardiac death, myocardial infarction, and musculoskeletal injury are rarely mentioned (Philipps et al., 2012). So exercise at a higher intensity than vigorous (hard) level, which referred to as very hard intensity, is not to be recommended as it could lead to an increase in various risks of morbidity and complications.

Intensity is “How hard the body needs to work to perform an activity “. It has two aspects 1) *Absolute Intensity*, the rate at which the body uses oxygen or MET (Metabolic equivalent) and 2) *Relative intensity* measured objectively as percent of maximal heart rate, percent of heart rate reserve, percent of VO2max and subjectively through Rate Perceive Exertion (RPE) self reporting as Borg scale (Borg, 1987), Visual Analogue Scale (VAS) and Likert scale (Philipps et al., 2012). These subjective indicators are named “subjecting rating scales” that imply the feeling of “what he thinks and is doing” and can provide 3 benefits as measurement, prescription and prediction for exercise intensity (Burkhalter, 1996).

Exercise higher than the moderate level is defined in Table 1 (Tipton et al., 2006), that shows vigorous (hard) intensity, has a Rate Perceived Exertion (RPE) on the Borg scale of 14-16. In case that RPE is higher than 16 the level of the exercise is very hard. Evidences of the exercise for health and benefits in metabolic syndromes obviously do not recommend exercise at the very hard level due to an increase the risk of mentioned adverse events, so the RPE of 16 was the upper limit of exercise prescription.

**Table 1:** Show relative intensity of exercise and RPE separated by levels of intensity from moderate to maximal

Intensity	%VO2 reserve or %Heart Rate reserve	%Heart Rate max	RPE
Moderate	40-59	55-69	12-13
Vigorous(Hard)	60-84	70-89	14-16
Very hard	>85	>90	17-19
Maximal	100	100	20

Many studies of exercise intensity based on subjective rating scales, including Borg scale, Visual analogue scale and Likert scale have been done to measure the level of pain and intensity of exercises such as the study by Harms et al. (1986) found no statically significance between the Borg scale and the VAS scale when using them to measure pain in the elbow of 8 people. Thus, we can use the Borg scale and Visual Analog Scale to assess the level of pain caused by the load weight on elbows. A study by Grant et al. (1999) tested Borg scale, VAS and Likert scale to assess the degree of exercise intensity at a vigorous level as 60-70% of VO2max, has found that the VAS is the most suitable subjective rating scale for reproducibility of breathlessness and general fatigue during exercise. VAS is also the most sensitive measure of the breathlessness while Borg scale is the most sensitive for assessing general fatigue. Capodaglio et al. (2001) investigated the relationship between VAS and Borg CR10 while doing an arm crank exercise and found that VAS increases in a linear relationship with workload. Whereas, the Borg CR10 (0 -10 fixed point-scale) found a slight increase together with workload and was significantly associated with heart rate and blood lactate. Ueda et al. (2006) studied the 4 scales of VAS (VAS 1-VAS 4) to measure the intensity of the exercise by both pedaling and running. The results support the 4-point scale of VAS as very useful to monitor the exercise intensity for both activities. Additional, results of the VAS1 and VAS2 for pedaling were statistically significantly higher than running, while at VAS3, subjects clearly felt leg pain greater than arm pain during arm cranks. At VAS4, running had breathing difficulty nearly the same as heart pain whereas pedaling had breathing difficulty much more than heart pain.

A study by Morris et al. (2007) compared the Verbal Numerical Rating Scale (VNRS) and VAS to assess the level of dyspnea in both younger and older subjects while performed treadmill walking. These 2 rating scales were used twice a day in the first and second day. The results stated that VNRS are reliable throughout the exercise and recovery, and that the VAS significantly decreased on the second day in accordance with the constant higher VNRS than VAS on subjects in both groups. Finally, it is appropriate to satisfactorily use both the VNRS and VAS as a Rating scale to measure symptoms of dyspnea.

Thus, the importance of exercise intensity in the prescription of exercise should always be consistent with duration and frequency, and is the first consideration for any subject especially in vigorous intensity exercise that needs a risk assessment of the individual, observations and surveillance for adverse events. So this brought about our motivation to study subjective rating scale during vigorous intensity exercise.

## 2. Objective

1) To investigate and evaluate 3 subjective rating scales, the Borg scale, the Combined Numerical Rating Scale (NRS) + FACES Dyspnea Rating Scale (FACES) and the Likert scale during the hard (vigorous) intensity exercise

2) To compare the effectiveness of the Borg scale and Combined Numerical Rating Scale (NRS) + FACES Dyspnea Rating Scale (FACES) during the hard (vigorous) intensity exercise.

## 3. Method

### 3.1 Sample

This study is a descriptive study, recruited participants in the health care allied professions, working and studying in Phramongkutklao Hospital. The sample size was calculated in accordance with the study of Grant et al. (1999) with an average level of VAS = 2.62 (SD=1.6) with 95% confidence and correlation at 25% (d = 0.65)


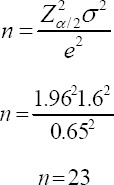


As n= sample size, α= 0.05 (Zα/_2_=Z_0.025_=1.96), e = the validity to estimate (equal to 25% of an average VAS) = 0.65, σ = the standard deviation of VAS = 1.6, then calculated for the sample size in this measurement of VAS, the minimum 23 participants is required. However, the data is insufficient to cross reference for the 3 methods. So, the calculations are based on the assumption of a minimum 23 participants in each group.

### 3.2 Inclusion Criteria

Participants fulfilling the following criteria were recruited

1) Studying or working in Phramongkutklao hospital, not restricted by gender or age
2) Passed a physical examination and history screening by a doctor, ECG. (Electrocardiogram), and no contraindications to the exercise criteria of The American College of Sports Medicine 2010
3) Leading an inactive lifestyle as defined by The American College of Sport Medicine 2010

### 3.3 Exclusion Criteria

1) On medications that affects heart rate, such as Beta blocker, Diuretics, Vasodilators etc
2) Engaging regularly in exercise for at least three day/week at moderate level

### 3.4 Settings

Participants had an appointment at Outpatient Department of Cardiology of Phramongkutklao hospital to do an Exercise Stress Test (EST).

### 3.5 Measurement

Participants were randomly placed into the 3 groups, the Borg scale, the Combined Numerical Rating Scale (NRS) + FACES Dyspnea Rating Scale (FACES), abbreviated to “NRS + FACES” and the Likert scale group.

**Figure 1:**
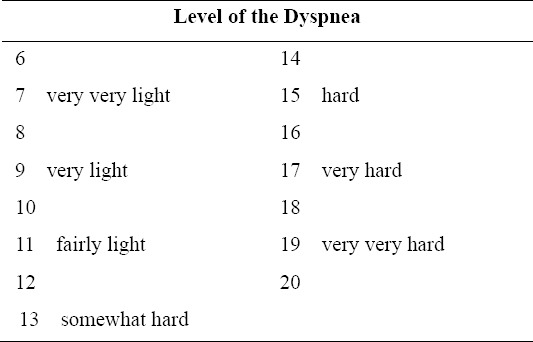
Borg scale or Rate Perceived Exertion (RPE)

**Figure 2:**
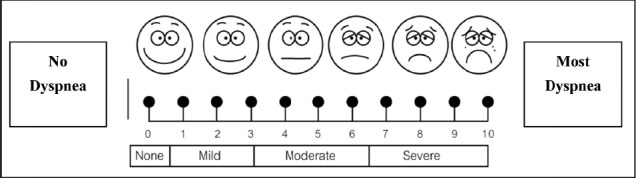
Numerical Rating Scale (NRS) plus FACES Dyspnea Rating Scale (FACES), NRS+FACES

**Figure 3:**
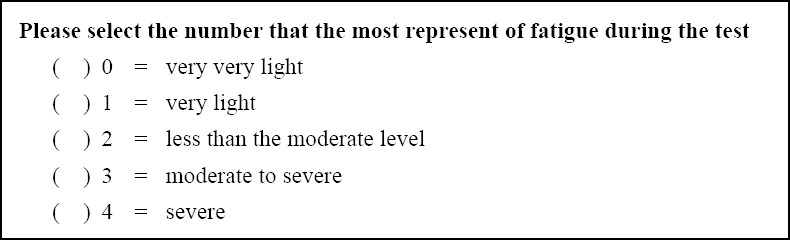
Likert scale

The original **Borg scale** ranges from 6-20 (odd range of 6-20), gradated on the odd numbers starting from 7 (very very light) to 19 (very very hard) (Figure 1). NRS + FACES are a combination of 1) the Numerical Rating Scale (NRS) between 0-10 (NRS11) = 11-point scale but substitutes the word Dyspnea for Pain. On the scale 0 = No Dyspnea, 1-3 = Mild Dyspnea, 4-6 = Moderate Dyspnea, 7-10 = severe Dyspnea and 2) the Wong Baker FACES Pain Rating Scale (FACES) (Tomlinson et al. (2010). This scale follows the NRS changing the word “pain” to Dyspnea. The “FACES Dyspnea Rating Scale (FACES)”. The Scale 0-10 is represented by 6 faces from left face to the right face at 1,2,3,4,5,6, equal to 0,2,4,6,8, and 10 respectively. Face number 1 on the left edge is the biggest smile, scale 0 = No Dyspnea and face number 6 on the right edge is the most grimacing, scale 10 = worst possible pain (Figure 2) (McCaffery et.al, 2011). The **Likert scale** is a Numerical Rating Scale, in which a level of agreement or disagreement of Dyspnea is recorded. It has a 5 alternative, quantitative scales range from 0-4 as details 0 = No Dyspnea, 1,2,3 and 4 mild, moderate, moderate to severe and severe Dyspnea, respectively (Figure 3).

### 3.6 Intervention (Figure 4)

**Figure 4:**
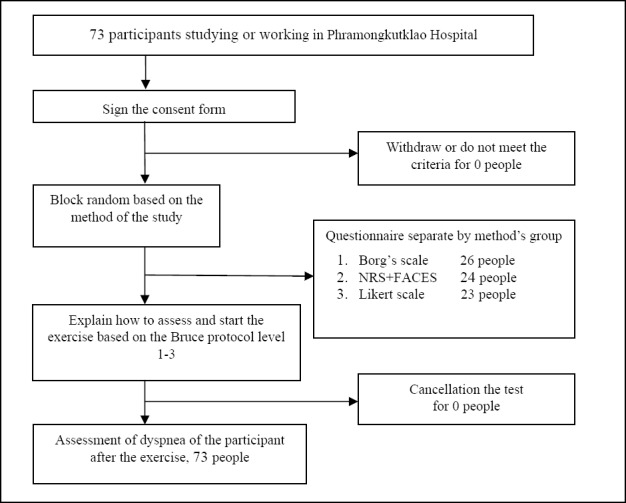
Flow Chart

1) Participants were informed by the researcher about research methodology and data gathering from the study. Any questions were asked and answered at this stage. After that, participants signed a consent form.
2) Participants were asked to answer the questions regarding, gender, age, underlying diseases, history of muscle and skeletal injury, duration since the last aerobic exercise until joining the research study, frequency of aerobic exercise undertaken in a week, and the type and duration of exercise.
3) Participants who agreed to take part in this study had a physical examination and Electrocardiography (ECG) by the doctor.
4) Participants were randomly assigned into 3 groups according to the type of assessment included Borg scale, NRS + FACES, Likert scale.
5) The research described some medical term such as, “Dyspnea” as being equal to “hard to breath” or “breathlessness”. The participants were also reminded not to include the feeling of faster breath, leg pain or fatigue misunderstood to be the feeling of dyspnea in each rating scales.
6). All of participants were explained how to monitor and assess dyspnea, warning signs or symptoms indicating to stop EST and the communication during exercise at target heart rate.
7). The demographic information including age, sex, nationality, height and weight was fed into the computer which was directly connected to an ECG machine to calculate the target heart rate during vigorous exercise, defined as **85%** of maximum heart rate of individual participant in the trial and a computer alarm sounded once the exercise reached the target level.
8) All of participants had a monitoring ECG lead to measure heart rate and blood pressure before starting the exercise on the treadmill which adjusted speed and slope automatically. The procedure used in this exercise was based on the Bruce protocol, which divides the speed into 3 levels which is directly associated with the intensity of the exercise (Thompson, 2010).
  - Level 1. Speed at 1.7 miles per hour and gradually increasing the slope to 10%
  - Level 2. Speed at 2.5 miles per hour and gradually increasing the slope to 12%
  - Level 3. Speed at 3.4 miles per hour and gradually increasing the slope to 14%
  All participants received an exercise level based on the Bruce protocol and received dyspnea evaluation form according to their assigned group. Once participants reached the target heart rate as calculated by the computer, the researcher will inform the participants to be aware so, the participants could make a mental note of their feelings of dyspnea so they could give their answers accurately in accordance with their assigned method of reporting, the Borg scale, the NRS+FACES, or the Likert scale.
9) Participants reported their feelings of dyspnea as soon as the walking exercise finished.

### 3.7 Statistical Analysis

Outcomes were analyzed using the SPSS software package on an intention-to-treat basis such as, number, percentage, and mean, standard deviation (SD), minimum and maximum. The comparison between the feeling of dyspnea during the exercise at target heart rate (85% of maximal heart rate), as assessed between Borg scale and NRS + FACE was analyzed by Chi-square to test the correlation between three methods and heart rate using the Spearman’s rank correlation coefficient to determine the significance at level of 0.05.

## 4. Results

There were 73 participants divided randomly into three groups using the Borg scale for 26 participants, NRS + FACES for 24, the Likert scale for 23. The average mean age of participants was 23.37 years old, 43 male, 30 female average weight 62.20 kg, average height 168.01 cms, average Body Mass Index 21.88kg/m^2^, average target heart rate during exercise, 169.70/min. The majority of the participants did not have underlying disease (Table 2).

**Table 2:** Show mean (SD) of participants’characteristics in 3 groups evaluated by Borg scale, NRS + FACES and Likert Scale

Characteristics of participants	Borg scale (n=26)		NRS+FACES (n=24)		Likert Scale (n=23)		Total (n=73)	
Age (yrs)	23.54	(3.43)	23.71	(3.36)	22.83	(3.51)	23.37	(3.41)
Sex (%)								
Male	16	(61.54)	14	(58.33)	13	(56.52)	43	(58.90)
Female	10	(38.46)	10	(41.67)	10	(43.48)	30	(41.10)
Weight (kg)	64.02	(12.30)	61.73	(12.27)	60.57	(8.17)	62.20	(11.13)
Height (cm)	170.31	(7.58)	166.88	(6.65)	166.55	(8.01)	168.01	(7.52)
BMI (kg/m^2^)	21.88	(2.62)	22.00	(3.22)	21.76	(1.81)	21.88	(2.60)
Target Heart Rate	169.8	(5.23)	168.67	(6.39)	170.57	(4.93)	169.70	(5.55)
Underlying disease (%)								
No	24	(92.31)	21	(87.50)	21	(91.30)	66	(90.41)
Yes	2	(7.69)	3	(12.50)	2	(8.70)	7	(9.59)

**Figure 5:**
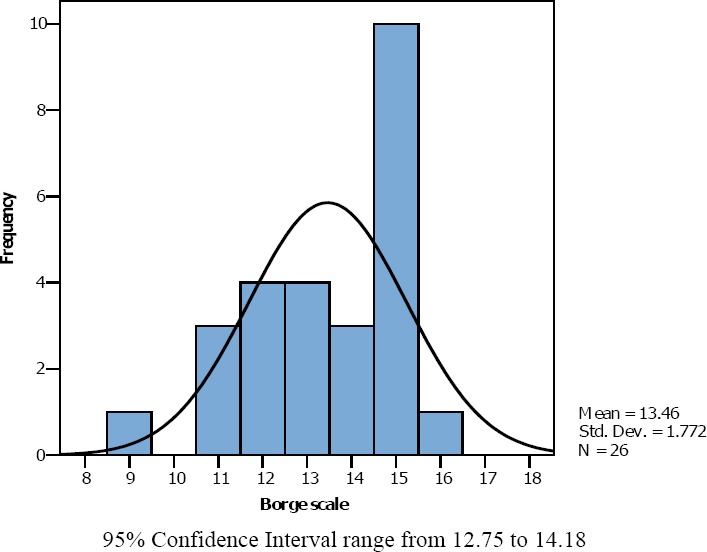
Borg scale assessment

**Figure 6:**
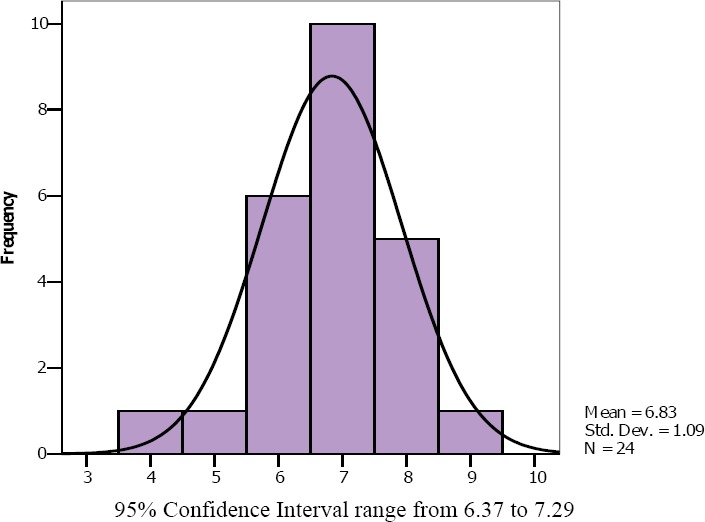
NRS + FACES assessment

**Figure 7:**
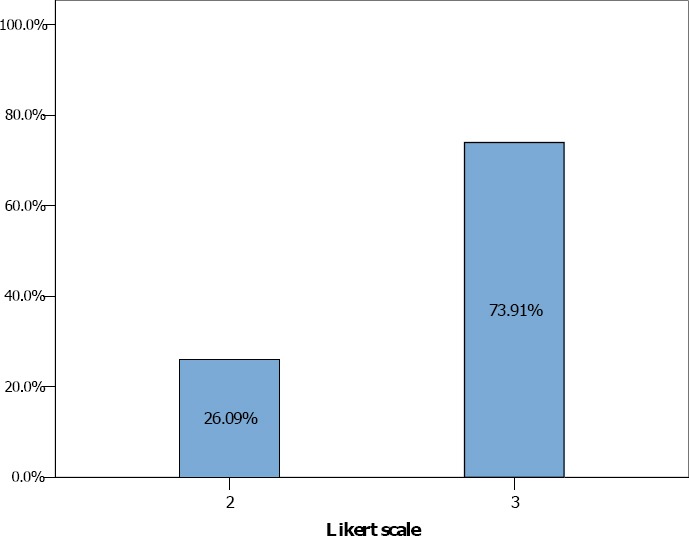
Likert scale assessment

In the Borg scale group (Figure 5), the average level of dyspnea (Mean Borg scale + SD) during vigorous (hard) intensity exercise was equal to 13.46 + 1.77. Regarding Mode, for Borg scale number 15 was the most selected by participants. On the other hand, the NRS + FACES group (Figure 6), the average level of dyspnea (Mean NRS + FACES + SD.) during vigorous (hard) intensity exercise was equal to 6.83 +1.09 and scale number 7 was the Mode. Finally, in the group that used Likert scale (Figure 7) a two-level scale, 6 participants endorsed level 2 (26.9%) and 17 choose level 3 (73.95%). So the average Likert scale (Mean) was 2.74.

A Comparison of the Borg scale and the NRS + FACES scale, as to the meaning of “hard or vigorous” during vigorous (hard) intensity exercise is shown in Table 3. In the Borg scale group participants with dyspnea equal to or higher than 15, that equals to “hard” for 11 participants (42.3%) and less than 15 for 15 patients (57.7%), while the NRS + FACES group participants had dyspnea equal to or higher than 7 which indicates severe dyspnea for 16 participants (66.7%) and less than 7 for 8 participants (33.3%). Therefore, comparing these 2 groups by Chi-square test, the results showed no statistical significant differences (p= 0.084).

**Table 3:** Show the comparison between participants assessed by Borg scale and those by NRS + FACES with hard (vigorous) feeling or more during hard (vigorous) exercise by Chi-square test

Scales	Less than Hard (Vigorous)	Hard (Vigorous) or more	P-value
Borg scale	15 (57.7%)	11 (42.3%)	0.084
NRS+ FACES	8 (33.3%)	16 (66.7%)	

Analysis of the relationship between the Subjective rating scales, the Borg scale, NRS + FACES and the Likert scale versus the target heart rate at 85% of maximal heart rate using Spearman’s rank correlation coefficient found it was -0.384, 0.97, and 0.38, respectively. However, there was no significant correlation across these three methods and heart rate at vigorous intensity exercise (Table 4).

**Table 4:** Show the association between the assessments of dyspnea and the heart rate at vigorous intensity exercise (85% of maximal heart rate)

Scales	Correlation	P- value
Borg’ scale	0.384	0.053
NRS+ FACES	0.97	0.654
Likert scale	0.38	0.864

## 5. Discussion

The participants had an average age between 22-23 years old, so youthfulness may have led to the less underlying disease, but would not be both the metabolic syndrome and musculoskeletal system that hinder or affect exercise.

NRS + FACES pain is a rating scale that often used to assess the subjective feeling of pain, especially in children. In the traditional model of Wong Baker FACES Pain Rating Scale (WBFPRS), which summarizes the results from various studies Tomlinson et al. (2010) found the advantages in WBFPRS more than in other faces pain rating scales, in that they included adequate psychometric properties, quick and simple to use, required minimal instruction, most preferred by children of any age, and having vivid changes which showed pain on the face. For the purpose of this study the tool was adapted to combine a numerical rating scale 0-10 as it could address pain feeling better than the original which only had 6 faces (Figure 2). Nevertheless, the traditional Borg scale is the dyspnea rating scale that has worldwide recognition (Borg, 1987). Borg scale has alternatively ever been used as a pain rating scale by Harms et al. (1986) who found that both Borg scale and Visual Analogue Scale can be truly used to assess the level of pain caused by the weight load on the elbow.

In other studies, VAS can alternately be used as the dyspnea rating scale, Rate Perceived Exertion and furthermore to find out its relationship with heart rate and work load. The study of Muza et al. (1990) revealed that VAS was close associated with Borg scale and the coefficient of variation was similar to the feeling of intensity of breathing during exercise in patients with chronic obstructive lung disease. Neely et al. (1992) found a significant correlation between VAS, BorgCR10 and blood lactate, heart rate during exercise with a bicycle ergometer in healthy subjects.

Capodaglio et al. (2001) found that VAS increased in a linear relationship with the increasing workload. The study of Ueda et al. (2006) found that 4-point VAS is exactly useful to monitor exercise intensity in both running and pedaling.

Table 3 showed the Borg scale and NRS + FACES such that when 85% of maximal heart rate was reached the 2 rating scales showed no significant differences. It is clear that the NRS + FACES scale was of such accuracy to measure exercise intensity as well as the Borg scale. Two results according with the study were Tangugsorn et al. (1998) who found in normal population that the 2 pain rating scales such as VAS and Borg scale can certainly be used to validate the severity of exercise and corroborated Grant et al. (1999) whose study showed the VAS had more sensitivity to measure changes in dyspnea than Borg scale significantly.

This study once used assessment of 3 rating scales at 85% of maximal heart rate. It did not evaluate each level of exercise intensity from mild (low), moderate intensity and more. However, the results of the Exercise Stress Test for all participants had been certified by a good experienced cardiologist and verified all normal hemodynamic responses without abnormal signs or symptoms.

Furthermore, the result also showed it was inappropriate to use Likert scale to assess exercise intensity or dyspnea rating because all of the participants (100%) were underestimated their real dyspnea, that was being at level 4 of the Likert scale. The reason may be the only 5-level-rating Likert scale that seemed to be inadequate to evaluate dyspnea thus this meant too much breadth of range of scale may lead to lower specificity of assessment.

As seen in Table 4, the 3 rating scales did not correlate with the target heart rate at 85% of maximal heart rate significantly. This may be due to the method of study not to evaluate the rating scales at any points of the vigorous intensity within the range of 70-89% of maximal heart rate, but only one point at 85% of maximal heart rate. Even though, rating at hard (vigorous) intensity range is truly also available from 14-16 in Borg scale level and NRS + FACES scale at 7-10. These may be the significant factors influencing the rating scales in this study not significantly correlate with heart rate.

Recommendations for future research are: 1) To address NRS + FACES at all levels of exercise intensity from low, moderate, hard (vigorous) and find out the correlation between NRS + FACES and heart rate response 2) To study the NRS (Numerical Rating Scale) compared with NRS + FACES at all levels of exercise intensity 3) To study the NRS and / or NRS + FACES compared with BorgCR10 at all levels of exercise intensity.

## 6. Conclusion

The use of NRS + FACES to assess the hard(vigorous) intensity exercise specified at 85% of maximal heart rate on treadmill walking saw no significant difference from the Borg scale (NRS + FACES at level 7 and the Borg scale at 15) whereas, the Likert scale is not sensitive enough to assess the intensity of dyspnea.
